# Exploration of potential novel drug targets for diabetic retinopathy by plasma proteome screening

**DOI:** 10.1038/s41598-024-62069-0

**Published:** 2024-05-22

**Authors:** Weichen Yuan, Wei Xu, Xin Xu, Bo Qu, Fangkun Zhao

**Affiliations:** 1 Department of Ophthalmology, The Fourth Affiliated Hospital of China Medical University, No. 102, Nanqi Road, Heping District, Shenyang, Liaoning China; 2 Key Lens Research Laboratory of Liaoning Province, Shenyang, China; 3 Department of Biochemistry and Molecular Biology, China Medical University, Shenyang, China

**Keywords:** Diabetic retinopathy, Mendelian randomization, Drug target, Plasma proteins, Drug discovery, Genetics, Medical research

## Abstract

The aim of this study is to identify novel potential drug targets for diabetic retinopathy (DR). A bidirectional two-sample Mendelian randomization (MR) analysis was performed using protein quantitative trait loci (pQTL) of 734 plasma proteins as the exposures and clinically diagnosed DR as the outcome. Genetic instruments for 734 plasma proteins were obtained from recently published genome-wide association studies (GWAS), and external plasma proteome data was retrieved from the Icelandic Decoding Genetics Study and UK Biobank Pharma Proteomics Project. Summary-level data of GWAS for DR were obtained from the Finngen Consortium, comprising 14,584 cases and 202,082 population controls. Steiger filtering, Bayesian co-localization, and phenotype scanning were used to further verify the causal relationships calculated by MR. Three significant (*p* < 6.81 × 10^−5^) plasma protein-DR pairs were identified during the primary MR analysis, including CFH (OR = 0.8; 95% CI 0.75–0.86; *p* = 1.29 × 10^−9^), B3GNT8 (OR = 1.09; 95% CI 1.05–1.12; *p* = 5.9 × 10^−6^) and CFHR4 (OR = 1.11; 95% CI 1.06–1.16; *p* = 1.95 × 10^−6^). None of the three proteins showed reverse causation. According to Bayesian colocalization analysis, CFH (coloc.abf-PPH4 = 0.534) and B3GNT8 (coloc.abf-PPH4 = 0.638) in plasma shared the same variant with DR. All three identified proteins were validated in external replication cohorts. Our research shows a cause-and-effect connection between genetically determined levels of CFH, B3GNT8 and CFHR4 plasma proteins and DR. The discovery implies that these proteins hold potential as drug target in the process of developing drugs to treat DR.

## Introduction

Diabetic retinopathy (DR), as a prevalent microvascular complication of diabetes, continues to be one of the primary reasons for avoidable vision loss in individuals of working age. Research suggests that among individuals with diabetes, the global occurrence of DR is estimated to be 34.6%, 6.96% for proliferative diabetic retinopathy (PDR), and 6.81% for diabetic macular edema (DME)^1^. The disease's pathology is intricate and encompasses numerous interconnected metabolic irregularities initiated by consistently elevated glucose levels, with early occurrence of augmented cytosolic reactive oxygen species (ROS) in the retina, and causes continued damage to the retinal tissues^2^. Currently, the main treatments of DR include panretinal photocoagulation, vitreoretinal surgery, anti-angiogenic drugs and intravitreal corticosteroids^3^. Although the aforementioned managements have significantly advanced the treatment of DME and PDR, some defects and limitations still exist, including high treatment costs, intricate clinical management and various complications. Furthermore, certain patients do not show favorable responses to these standard-of-care approaches. A study showed that, persistent diabetic macular edama (pDME) following three monthly injections at week 12 varied at rates of 50.8%, 72.9%, and 53.2% for eyes treated with aflibercept, bevacizumab, and ranibizumab, respectively^4^. Approximately 12% of patients who underwent laser treatment still experienced vision loss^5^. Therefore, development of new drug targets, biomarkers and their clinical translation are essential and highly cost-effective for the management of DR^6^)

Human proteins, such as immunoglobulins and cytokines, are the predominant types of drug target and play crucial roles in immune responses and defense mechanism. Immune dysfunction has been consistently observed in individuals with DR^7–9^, while previous studies showed that plasma proteins is closely related to immunologic derangement^10^. Although the exact causes and mechanisms behind these immune disturbances are not yet well understood, the plasma proteomics offers a unique opportunity to explore this question. Studying the link between genetic susceptibility to DR and plasma proteins may be a crucial stride in comprehending the fundamental causes of immune dysfunction and identifying potential novel drug targets.

Mendelian randomization (MR) is a promising method for drug target identification by exploiting genetic variants associated with exposures or risk factors to estimate possible causal relationships to outcomes. The objective of this method is to reduce the influence of confounding factors and reverse causality that could distort the results in epidemiological investigations^11^. With advances in high-throughput genomic and proteomic technologies in plasma and cerebrospinal fluid, the use of MR-based strategies to identify potential therapeutic targets has become easier by integrating genome-wide association studies (GWAS) and protein quantitative trait loci (pQTL). At present, this MR method has been widely recognized and has been used to identify therapeutic targets for multiple sclerosis, asthma, small cell lung cancer and other diseases^12^. In addition, protein drug targets with genetic support and guided selection were found to present a higher probability of receiving market approval, twice as much as those lacking such support according to a prior investigation^13^. To our knowledge, there have been no MR study that have integrated GWAS and pQTL data in relation to DR. Hence, the aim of this research is to fill this void by initially exploring plasma proteins as possible targets for drugs and indicators for DR through the combination of extensive GWAS and pQTL data using MR analysis.

## Methods

### Plasma protein quantitative trait loci selection

**Figure 1:**
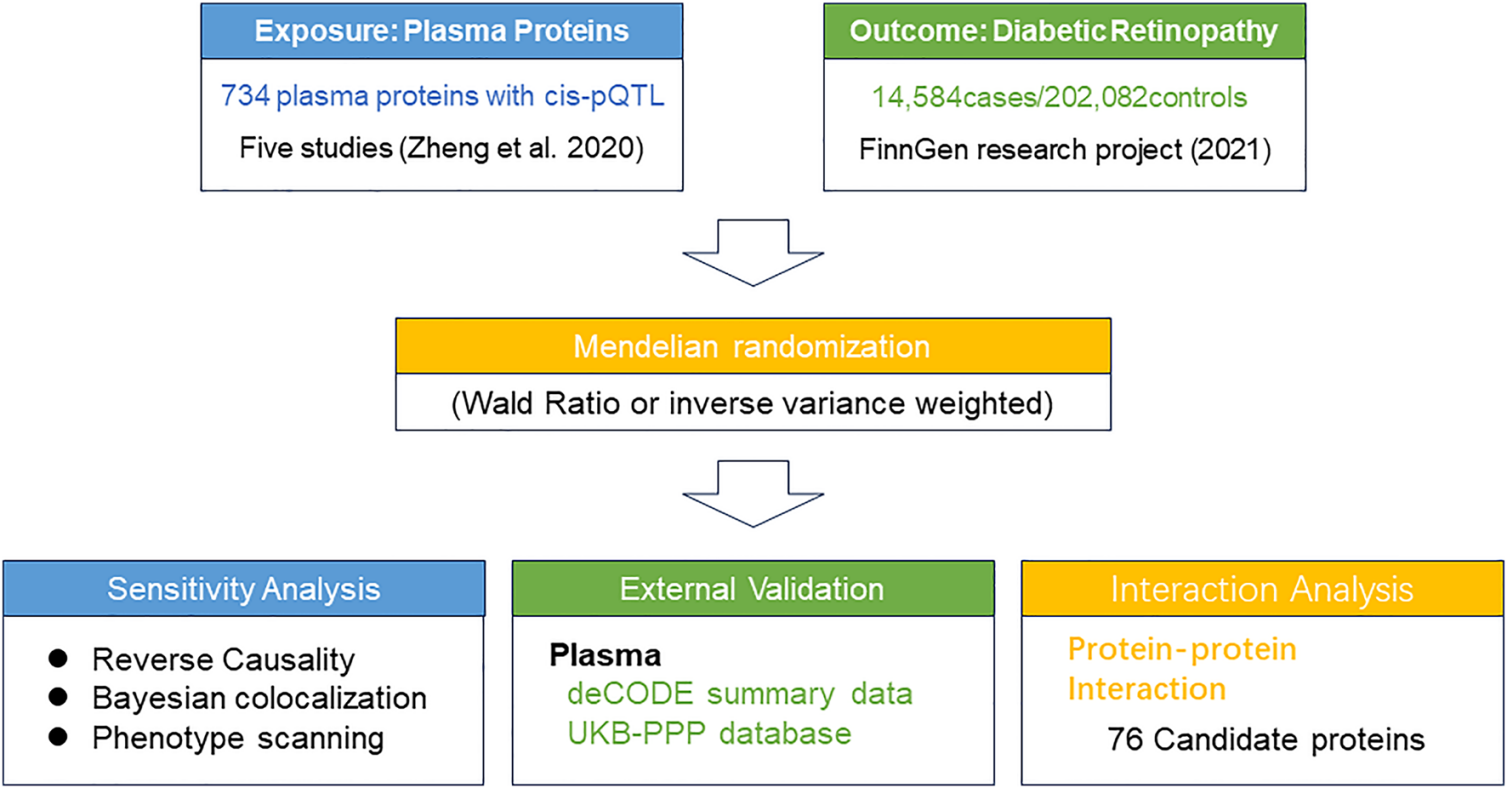
Flowchart of identifying causal plasma proteins for diabetic retinopathy by Mendelian randomization.

The plasma pQTL information employed in the main analysis was obtained from a previous study^14^, which integrated data from five GWAS studies that were published earlier^15–19^. Only pQTLs meeting the following requirements were considered: (i) having a genome-wide significant association (P < 5 × 10^−8^), (ii) being situated outside the major histocompatibility complex (MHC) region (chr6, 26–34 Mb), (iii) demonstrating independent association [linkage disequilibrium (LD) clumping r^2^ < 0.001)], and (iv) being cis-acting pQTL. Based on the screening criteria used above in the plasma pQTL dataset, 738 cis-acting single nucleotide polymorphisms (SNPs) for 734 proteins were included. The detailed information of SNPs used as instrumental variables for each pQTL including their F-statistics is shown in Supplementary Table 1. Furthermore, the plasma pQTL data obtained from Ferkingstad et al.^20^ and Sun et al.^21^ were used for external validation. The original file was carefully verified for plausibility. To address any missing information in the pQTL GWAS summary statistics, such as effect allele frequency, the 1000 Genomes Project European samples were used as a reference to complete the data. (grch37.ensembl.org/Homo_sapiens/Info/Index) The flow chart of this MR analysis was shown in Fig. 1.

### GWAS summary statistics of diabetic retinopathy

In order to minimize bias caused by sample overlap, the summary statistics for the DR GWAS were obtained from the FinnGen research project (https://r5.finngen.fi/), comprising 14,584 cases of DR and 202,082 population control cases. DR in this study was defined according to the International Classification of Disease-10.

### MR analysis and external validation

For this study, we performed MR with plasma proteins as exposures and DR as the outcome. The analysis was conducted using the R programming language (version 4.3.1) with the TwoSampleMR R package (Version 0.5.6). In cases where there was only one available pQTL for a particular protein, we utilized the Wald ratio. When there were multiple genetic instruments available, we performed inverse variance weighted MR (MR-IVW) followed by heterogeneity analysis^22^. The increased risk of DR was quantified using odds ratios (ORs) represented as the standard deviation (SD) of the rise in plasma protein concentrations.

We utilized a strategy that employed the same SNP used in the primary analysis as genetic instruments, along with a strategy that utilized genome-wide significant SNP as genetic instruments to validate initial findings. The detailed pQTL data used for external validation were shown in Supplementary Table 2. During the initial analysis, we utilized the Bonferroni correction method to account for multiple testing. We established a threshold p-value of 0.05/734 (*p*-value < 6.81 × 10^−5^) to identify potentially significant causal proteins. Afterwards, these proteins were employed for external validation with a significance level of 0.05.

### Reverse causality analysis

To detect reverse causality in our main MR analysis, we performed bidirectional two-sample univariable MR and employed Steiger filtering. Thirteen genetic instruments that were significantly valid for DR were identified from the FinnGen research project (Supplementary Table 3). We also obtained comprehensive summary statistics for proteins from two previous studies^16,20^. To determine the associations between proteins and DR, a MR analysis was conducted using MR-IVW, MR-Egger, weighted median, simple mode and weighted mode. Steiger filtering was used with a significance threshold of P < 0.05 to ascertain the directionality of the association, as a supplement.

### Bayesian co-localization analysis

To estimate the probability of two traits sharing a common causative variable, we employed the ‘coloc’ R package (Version 5.1.0) and its predefined parameters for Bayesian collocation analysis^23^. For each locus, the Bayesian approach assessed five different hypotheses: (1) no connection with either trait, (2) connection with only trait 1, (3) connection with only trait 2, (4) both traits are linked to different causal variants, and (5) both traits are linked to the same causal variant^24^. After the analysis, posterior probabilities were obtained for all the hypotheses including H0, H1, H2, H3, and H4. We determined that two signals exhibited substantial evidence of colocalization when the posterior probability for shared causal variants (PPH4) was equal to or greater than 0.8. A medium level of colocalization was defined as PPH4 being between 0.5 and 0.8^25^.

### Phenotype scanning

To search for associations between the identified pQTLs and other characteristics, we conducted phenotype scanning. (https://gwas.mrcieu.ac.uk/phewas/) The pQTLs that satisfied the given conditions were deemed to possess pleiotropic impacts, demanding cautious analysis of their consequences: (1) the presence of a significant association at the genome-wide level, denoted by *p* < 1 × 10^−5^; and (2) the pQTLs exhibiting links with established risk factors pertaining to DR, such as proteins, genes, or diseases. In addition, we calculated the LD r^2^ among pQTLs of prioritized proteins to reveal potential linkage.

### Protein–protein interaction network analysis

In the plasma analysis, we investigated the protein–protein interaction (PPI) network of proteins that may be associated with a higher risk of DR (primary MR analysis *p* < 0.05). The goal was to study the connections between the identified proteins. For all PPI analyses, we used version 11.5 of the Search Tool for the Retrieval of Interacting Genes (STRING) database (http://string-db.org/), with a minimum interaction score of 0.4^26^.

### Ethics approval and consent to participate

All analyses were based on publicly shared databases and no additional ethical approvals were required.

## Results

### Screening the proteome for diabetic retinopathy causal proteins

**Figure 2:**
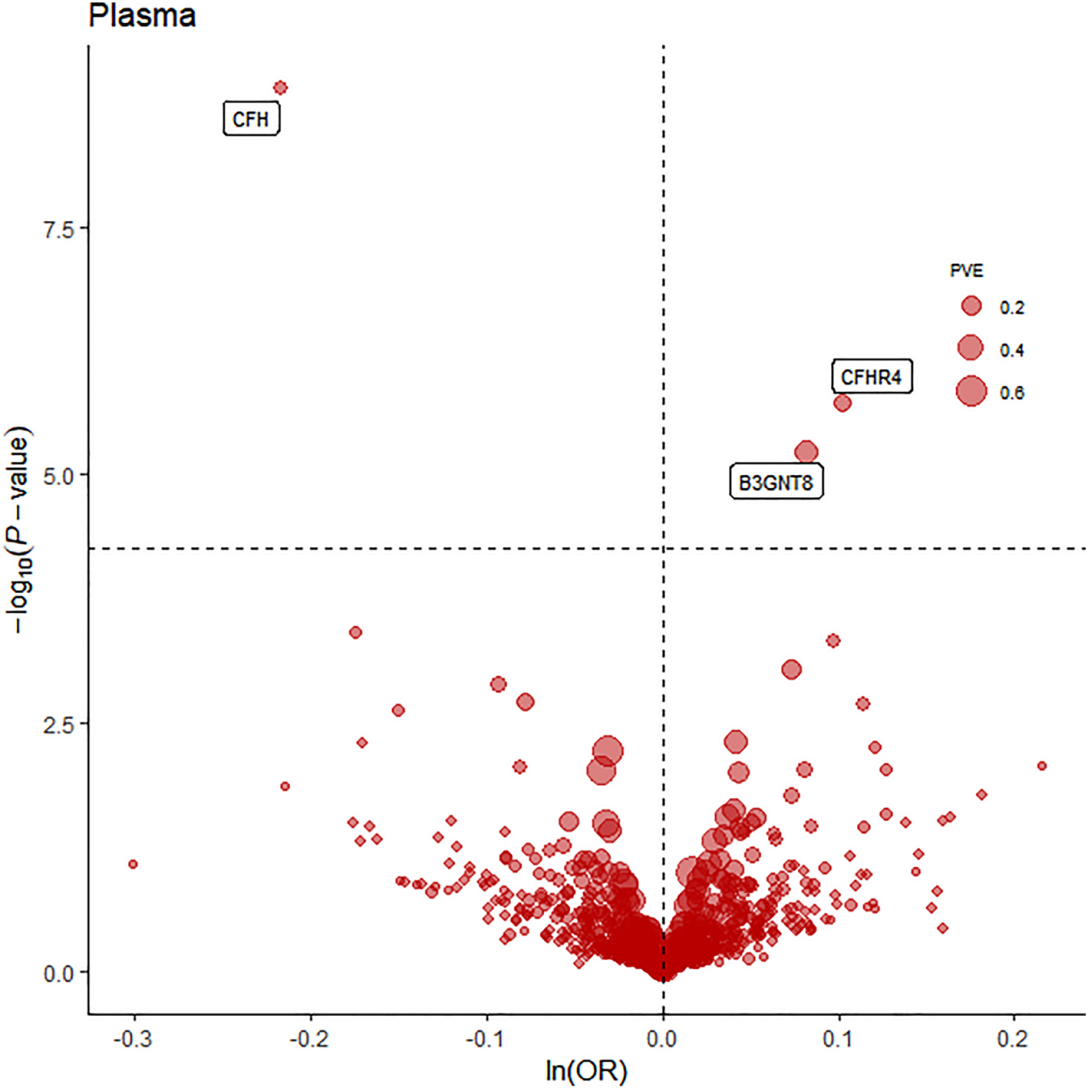
Volcano plot of the MR analysis for 734 plasma proteins on DR risk. *OR* odds ratio, per standard deviation increase in plasma protein levels. Dashed horizontal line represented p-value = 6.81 × 10^−5^. *PVE* proportion of variance explained.

During the primary MR analysis, three notable plasma protein-DR pairs were identified (*p*-value < 6.81 × 10^−5^; Fig. 2 and Table 1), which encompass complement factor H (CFH), β-3-N-acetylgluco-saminyltransferase 8 (B3GNT8), complement factor H-related protein 4 (CFHR4). In particular, a rise in CFH (OR = 0.8; 95% CI 0.75–0.86; *p* = 1.29 × 10^−9^) was correlated with a reduced likelihood of DR, while higher levels of B3GNT8 (OR = 1.09; 95% CI 1.05–1.12; *p* = 5.9 × 10^−6^) and CFHR4 (OR = 1.11; 95% CI 1.06–1.16; *p* = 1.95 × 10^−6^) increased the risk of DR. Moreover, there was no heterogeneity observed in relation to the examined plasma proteins.

**Table 1:** MR results for plasma proteins significantly associated with DR after Bonferroni correction.

Protein	UniProt ID	SNP	Effect allele	OR (95% CI)	*p* value	PVE	*F* statistics	Reference
CFH	P08603	rs2274700	A	0.80 (0.75, 0.86)	1.29e–09	6.45%	227.48	Sun et al.
B3GNT8	Q7Z7M8	rs284663	T	1.09 (1.05, 1.12)	5.90e–06	28.17%	1255.18	Emilsson et al.
CFHR4	Q92496	rs4915559	T	1.11 (1.06, 1.16)	1.95e–06	16.55%	63465	Emilsson et al.

*PVE* proportion of variance explained.

### Sensitivity analysis for diabetic retinopathy causal proteins

At first, the bidirectional analysis of MR did not discover any causal impact of DR on the concentrations of the three identified proteins, and the directionality was further confirmed by Steiger filtering (Table 2). Furthermore, according to Bayesian colocalization analysis, CFH (coloc.abf-PPH4 = 0.534) and B3GNT8 (coloc.abf-PPH4 = 0.638) in plasma may share the same variant with DR mediumly (0.5 < PPH4 < 0.8). Finally, the phenotype scanning uncovered connections between CFH (rs2274700) and age-related macular degeneration (AMD), lung function, B3GNT8 (rs284663) and height, coronary artery illness, basal metabolic rate, as well as CFHR4 (rs4915559) with neovascularization and AMD. The all proteins and phenotypes related to CFH (rs2274700), B3GNT8 (rs284663) and CFHR4 (rs4915559) were showed in Supplementary Table 4.

**Table 2:** Summary of reverse causality detection, Bayesian co-localization analysis and phenotype scanning on three potential causal proteins

Protein	UniProt ID	SNP	Bidirectional MR, OR (95% CI)	Steiger filtering	Co-localization PPH4 (coloc.abf)	Previously reported associations
CFH	P08603	rs2274700	0.99 (0.93–1.05)	TRUE (6.98 × 10^–45^)	0.534	AMD, lung function,
B3GNT8	Q7Z7M8	rs284663	1.25 (0.67–2.33)	TRUE (8.12 × 10^–234^)	0.638	Height, CAD, basal metabolic rate
CFHR4	Q92496	rs4915559	1.01 (0.9–1.14)	TRUE (77.3 × 10^–124^)	1.81 × 10^–5^	Neovascularization, AMD

*NA* not available, *CAD* coronary artery disease, *AMD* age-related macular degeneration.

### External validation of potential drug targets for diabetic retinopathy

**Figure 3:**
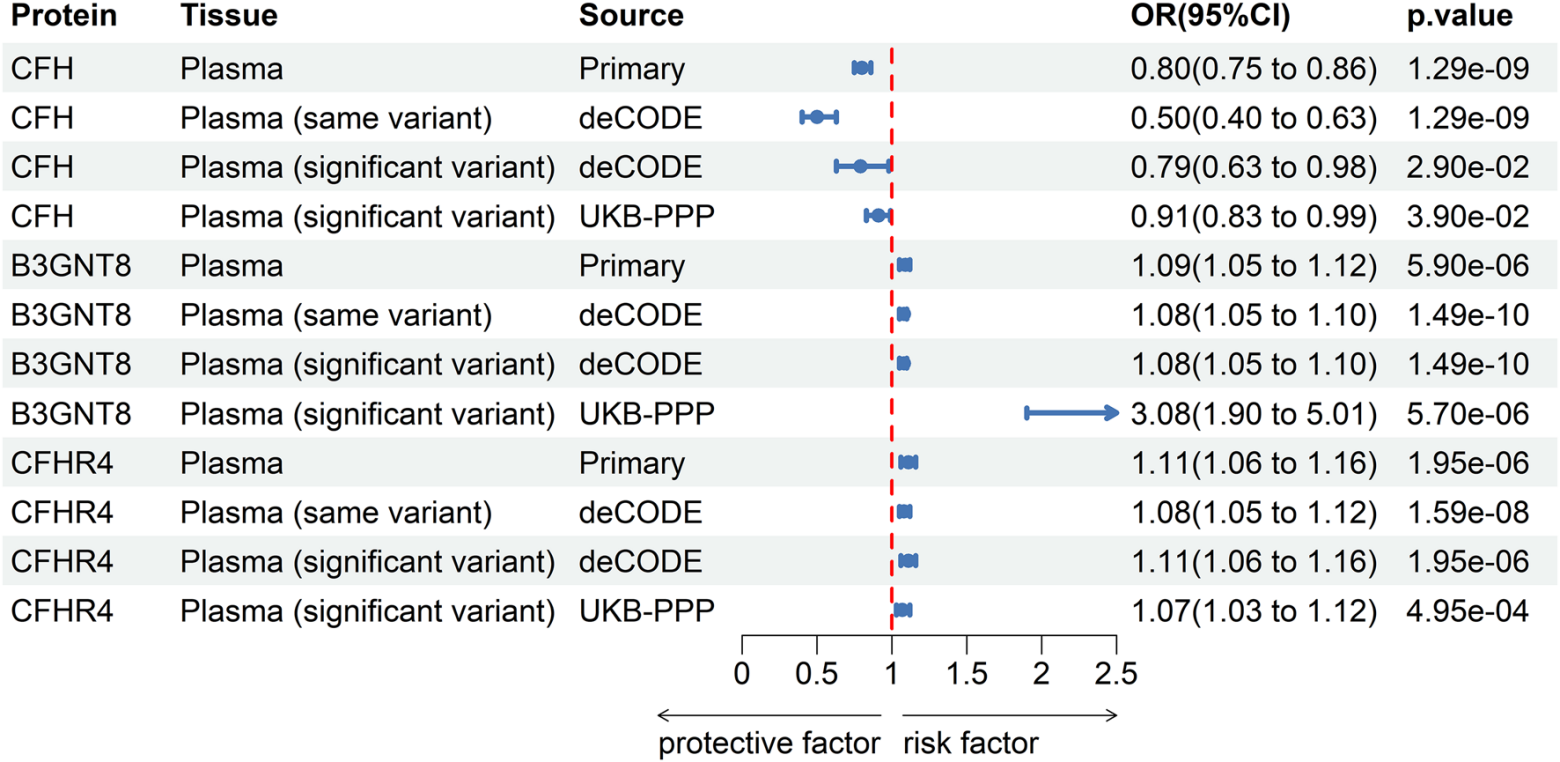
External validation of the causal relationship between diabetic retinopathy and three potential causal plasma proteins by Mendelian Randomization analysis.

During the external validation, the same-variant and significant-variant approaches were utilized to reaffirm the casual impact of three plasma proteins discovered in the initial analysis, showing that CFH, B3GNT8 and CFHR4 are associated with DR (Fig. 3). For each protein, consistent results were observed for all SNPs (primary, same-variant and significant-variant). For example, the increased amount of CFH consistently lowered the likelihood of DR across the three types of SNPs employed (primary: OR = 0.92, 95% CI 0.85–0.98, *p* = 0.018; same variant: OR = 0.86, 95% CI 0.76–0.97, *p* = 0.018; significant variant: OR = 0.86, 95% CI 0.76–0.97, *p* = 0.018).

### Protein–protein interaction network analysis

**Figure 4:**
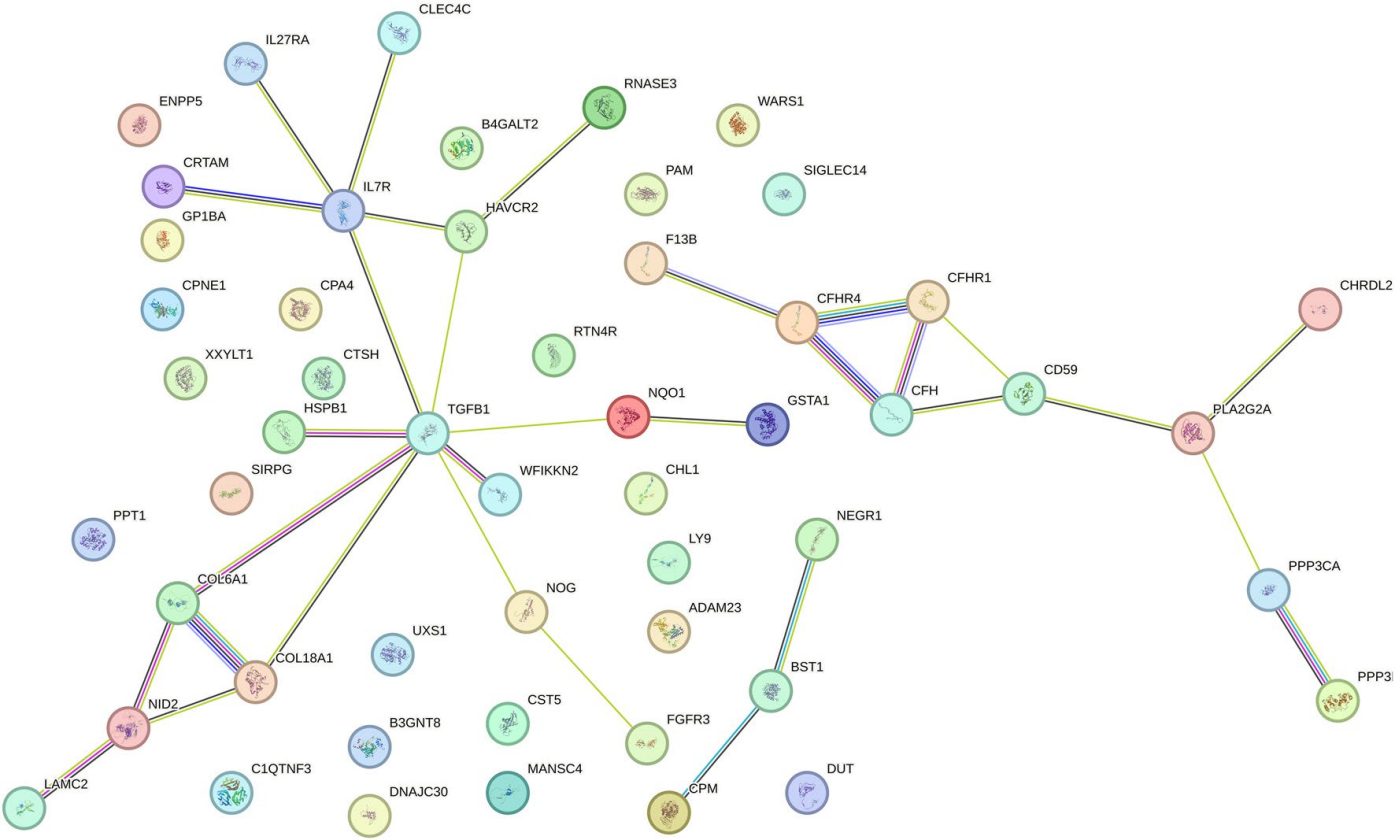
Protein–protein interaction network among the suggestive causal proteins (P < 0.05).

We performed a PPI analysis on the 76 identified plasma proteins responsible for causation. Figure 4 demonstrates STRING network with medium model confidence (0.400), which shows 52 nodes in the network with 31 edges (interactions) and has significantly more than the 10 expected interactions in a randomly selected set of proteins the same size (*p*-value < 9.18 × 10^–8^). The findings indicated that CFH seems to be associated with CFHR1 and CD59 through text mining and co-expression, and CFH may also be linked to CFHR4 through protein homology, gene co-occurrence, co-expression, and test mining. Furthermore, there could be a potential association between CFHR4, coagulation factor XIII B chain (F13B) and CFHR1. B3GNT8 had no association with other proteins uniquely. Meanwhile, CFH and CFHR4 pQTLs showed a suggestive correlation (LD r^2^ = 0.36) based on European population.

## Discussion

According to our understanding, this study is the initial attempt to utilize extensive proteome datasets in investigating causal proteins for DR through the application of two-sample MR and Bayesian co-localization. Through initial examination and external verification, we have identified three proteins, namely circulating CFH, CFHR4 and B3GNT8, that show promise as potential targets for DR treatment. A ‘causality identified’ by MR may be attributed to reverse causality, horizontal pleiotropy, or genetic confounding caused by LD. Therefore, the study included bidirectional MR analysis and found no proteins identified by the initial MR analysis that exhibited reverse causality, the finding that was additionally confirmed through Steiger filtering. Furthermore, Bayesian co-localization was employed to eliminate the bias caused by LD. CFH and B3GNT8 were likely to share the same variant of DR. Given the failure of co-localization of CFHR4 and the suggestive association between CFH and CFHR4 (LD r^2^ = 0.36), we speculated that the effect of CFHR4 in plasma might be a proxy of CFH’s effect. Also, our research revealed that the protein targets were associated with different characteristics by means of phenotype scanning. The findings indicated a correlation between CFHR4 (rs4915559) and AMD, suggesting a potential shared etiology between AMD and DR.

At present, the treatments of DR mainly include Anti-VEGF intraocular injections, sustained-release corticosteroid injections, laser therapy and vitrectomy^3^. Various adverse ocular reactions have been documented when using Anti-VEGF injections, which consist of subconjunctival hemorrhage, vitreous hemorrhage, inflammation within the eye, endophthalmitis, elevated intraocular pressure (IOP), and retinal detachment. In addition, it is worth noting that Anti-VEGF treatment may carry some systemic risks, although the likelihood of occurrence is relatively low^27^. Moreover, due to the short effective duration of Anti-VEGF drugs, patients with DR usually need to rely on continuous intravitreal Anti-VEGF injections to treat DR. However, due to factors such as financial constraints, lack of patient compliance, and the unavailability of timely treatment (e.g. during the peak of the COVID-19 pandemic), patients may experience a decline in clinical condition or be unable to receive regular care^28^. Moreover, Anti-VEGF therapy is not always effective, according to the findings of DRCR.net Protocol I, approximately 40% of eyes treated with ranibizumab for DME showed incomplete responses and suboptimal vision outcomes within a three-month period^29^. Intravitreal corticosteroids work on various pathways associated with the formation of DME in order to decrease the effects of inflammatory cytokines and VEGF, lower leukostasis, and enhance other physiology. However, as with Anti-VEGF therapy, it may not be timely for routine clinical management and may lead to elevated IOP and the onset of cataract^30^. Laser therapy offers benefits such as diminishing the need for multiple injections and delivering effective and enduring treatment outcomes^31^. Nevertheless, the best corrected visual acuity may not be as good as the combination of Anti-VEGF treatment in some cases, and there may be side effects and adverse permanent visual consequences associated with the use of laser photocoagulation alone, such as the formation of scotomas, choroidal neovascularization, retinal scars and patient discomfort^32^. While vitreoretinal surgery is usually used for advanced complications of PDR, it is expensive, has low economic benefit, sometimes has limited effect on visual recovery, and has the risk of complications like vitreous hemorrhage, iatrogenic retinal damage and neovascular glaucoma^33,34^. Therefore, the existing treatment methods, whether surgery or drug targets, have certain defects, and the development of new drug targets is urgently needed. As mentioned above, we screened three novel plasma protein, namely CFH, CFHR4 of the same gene cluster as CFH, and B3GNT8 and they could be potential drug targets for DR treatment.

CFH is an essential negative regulator of complement activation via the alternative pathway and the C3b amplification loop, which plays a crucial role in regulating the alternative pathway C3 convertase. CFH acts as a brake device in the alternative pathway and effectively controls complement activation. This regulatory system prevents excessive complement activation and minimizes harm to the body^35,36^. CFH is located on chromosome 1q32. Currently, in the field of ophthalmology, the common SNPs in CFH are genetically strongly associated with AMD^37–40^, but no study of CFH on pathological progress of DR in specialty. Besides AMD, sequence and copy number variants in the human CFHR gene cluster have been associated with kidney disease Atypical hemolytic uremic syndrome (aHUS), C3 glomerular disease, IgA nephropathy (IgAN), and infection^41^. The distinct functions of the individual CFHR proteins within the entire cascade indicate that individuals with CFH gene abnormalities might find therapeutic value in one of the emerging complement inhibitor and clinical trials of complement therapy for glomerular diseases are also underway^42^. In the development of ophthalmic drugs, AVD-104 is the first drug targeting CFH as a therapeutic target. It is currently undergoing clinical trials and can activate CFH to bind with C3 with higher affinity, leading to the degradation of C3 convertase. This is considered a promising therapeutic target for dry AMD^43^. Unfortunately, there are no human studies specifically focused on the use of complement therapy for DR currently^48^. Studies have indicated that there was a noteworthy elevation in C3 and its activated fragment C3bα' (110 kDa) in the vitreous of patients with PDR. This was accompanied by an upregulation of CFH, which validated the enhanced activation of the alternative complement pathway in PDR. It is worth discussing that in our study, CFH, which serves as a protective factor for DR, unexpectedly showed a significant upregulation in the research conducted by Shahulhameed et al. They proposed that this might be a feedback mechanism maintaining the level of C3bα’ in the vitreous body of PDR and could be related to the activation of microglial cells^44^. We estimate that the phenomenon of increased CFH levels may be related to the risk variants of CFH, with mechanisms similar to AMD. No specific type of SNP for CFH was confirmed in their study, so our speculation cannot be ruled out. In comparison to NPDR cases, the serum CFH levels in PDR cases showed a downward trend, suggesting that CFH may play an important immunoprotective role in the early development of DR, leading to a compensatory increase, while its immunoprotective role is relatively suppressed in the progression to PDR. However, increasing evidence suggests that diabetes affects the entire retinal neurovascular unit, leading to neurodegeneration, followed by clinical vascular changes, which are early pathological processes in DR^45^. Furthermore, according to Fig. 4, our PPI results demonstrate that CFH seems to be associated with CD59 through text mining and co-expression. Studies have shown that CD59 is associated with DR. In the retinal tissues of DR patients, the expression of CD59 decreases. In the retinas of DR animal models, silencing CD59 leads to aggravated retinal vascular leakage. This further indicates that the interaction between CFH and CD59 may jointly affect the pathogenesis of DR. Therefore, CFH as a potential target for complement therapy is promising and may play a key role in the pathological progression of DR.

CFHR4 is one of the five complement factor H-related proteins in the CFH family (CFHR1/2/3/4/5). Consistent with the high genetic correlation between CFH and AMD, the SNP rs6685931 in CFHR4 and its associated haplotype H1-2 also confer a risk for AMD development^46^. CFHR4 accumulates in the choriocapillaris, Bruch’s membrane and drusen, and can compete with FH/FHL-1 for C3b binding, preventing FI-mediated C3b cleavage. The protective allele of the strongest AMD-associated CFH locus variant rs10922109 has the highest association with reduced CFHR4 levels (*p*-value = 2.2 × 10^–56^)^47^. Besides AMD, numerous studies have suggested that CFHR4 plays a role in immune system disorders such as systemic lupus erythematosus^48^, aHUS^49^, and hepatocellular carcinoma (HCC)^50^. Nonetheless, the research conducted on CFHR4 has been limited, with only a few studies available. So far, no studies have successfully determined the specific role of CFHR4 in the development or progression of DR. In the study conducted by Hongjun et al., the use of Gene Set Enrichment Analysis (GSEA) was used to reveal a correlation between differential CFHR4 expression and FCGR, PLK1, MAPK, ATR, MCM, PI3K, FGFR1, and FOXM1 pathways in patients with HCC^50^. FCGR, MAPK, PI3K and FGFR1 pathways are related to DR, so we speculate that differential expression of CFHR4 is also related to DR. In the study by Wu et al., FCGR3A was identified as a potential biomarker for patients with PDR. FCGR3A is a member of the FCGR family of plasma proteins^51^. MAPK-related pathways are currently a hot topic in DR research. The present study provides evidence that the activation of p38 MAPK is involved in the long-term vascular changes seen in DR. By inhibiting the activation of p38 MAPK in diabetic conditions, the death of both endothelial cells and pericytes, as well as the degeneration of retinal capillaries, was significantly prevented^52^. Moreover, human umbilical cord-derived mesenchymal stem cells provide protection to the vascular endothelium against diabetic damage and this effect is achieved through a paracrine mechanism mediated by the MAPK/ERK signaling pathway^53^. Naturally, PI3K is also one of the hotspots in DR research. Inhibiting the PI3K/Akt/mTOR signaling pathway can suppress high glucose-induced endothelial-mesenchymal transition^54^, and by inhibiting PTEN expression, the PI3K/Akt/VEGF signaling pathway can be activated, stimulating the activity and angiogenesis of retinal vascular endothelial cells (RVEC) in DR rat^55^. In Tang, et al., study, inhibiting the PI3K/Akt/Stat3/NF-κB signaling pathways can be effective in treating experimental DR^56^. Fibroblast growth factor receptor 1 (FGFR1) is also believed to play a crucial role in DR. High glucose can induce the activation of FGFR1-coupled MAPK in Müller glial cells, further enhancing the production of VEGF by activated Müller glial cells^57^. Inhibiting FGFR1 can improve VEGF-induced neovascularization and vascular permeability^58^. In summary, as a risk factor against DR, CFHR4 could potentially become an important therapeutic target in future research extensively. However, further verification is needed for its differential expression in DR, and whether CFHR4 can really affect these DR-related signaling pathways also remains to be confirmed.

B3GNT8 is a member of the B3GNT family and functions as an enzyme. Its role involves transferring *N*-acetylglucosamine (GlcNAc) to galactose residues present at the nonreducing end of poly-*N*-acetyllactosamine structures. B3GNT8 is primarily involved in extending specific branches of multiantennary *N*-glycans^59,60^. The mRNA expression of B3GNT8 is found in glycinergic amacrine cells of the retina^61^. Currently, research on B3GNT8 is mainly focused on the field of cancer. It is an important protein in the study of tumor drug resistance. Silencing B3GNT8 can improve multidrug resistance in human leukemia^62^ and improve resistance of colorectal cancer (CRC) to 5-Fu^63^. Unluckily, B3GNT8, as a risk factor related to DR we have identified, has not been studied in ophthalmology. But the important role of B3GNT8 in the *N*-acetylglucosamine process makes it a potential key target in the pathological process of DR. Existing studies have shown a close association between *N*-glycans and DR, the concentration of *N*-glycans in the vitreous was found to be significantly higher in patients with DR compared to those without diabetes^64^. Additionally, it was observed that there was an increase specifically in sialylated *N*-glycans in the vitreous fluid of patients with DR^65^. As an important modification pathway of *N*-glycans, GlcNAc may also play an important role in the pathological process of DR. *O*-GlcNAc modification refers to a type of protein glycosylation that stands out from other common forms due to its dynamic cycle. Its specificity for Ser/Thr residues and capability to bind with cytoplasmic and nuclear proteins is a particular form of GlcNAc^66^. Imbalanced *O*-GlcNAc modification may involve the etiology of diabetes and the pathogenesis of various diabetes complications^67^. *O*-GlcNAcylation is also a post-translational modification process and has been found to impact the selection of mRNAs for translation and facilitate mitochondrial dysfunction in the retina. Specifically, it promotes the involvement of 4E-BP1, a protein that plays a role in regulating the initiation of protein synthesis, in this mitochondrial dysfunction^68^. Moreover, hyperglycemic conditions are harmful to various tissues and cell types. Cells particularly affected is the retinal vascular cells, specifically the pericytes (PC), which experience early loss under these conditions. The post-translational *O*-GlcNAc modification of p53 and its elevated levels could possibly play a role in the specific early loss of PC observed in DR^69^. In conclusion, B3GNT8 may regulate *O*-GlcNAc and thereby affect important physiological processes related to DR, such as cell apoptosis, inflammation, and metabolism. Therefore, it could be a promising target for new drug therapies for DR. Further identification of *O*-GlcNAc modified targets and the function of B3GNT8 to *O*-GlcNAc modification will help us understand the molecular characteristics of DR. Since there are no relevant studies on B3GNT8 in DR research currently, further study on *O*-GlcNAc and B3GNT8 in DR is urgently needed.

This study has several significant strengths. This is the first article to use MR analysis to explore potential drug targets for DR, which minimizes bias caused by reverse causation and confounding factors. At the same time, the sample size included in this study is larger than that of traditional observational studies and has higher statistical power. Moreover, external validation of replicate cohorts is not a routine practice in MR studies. We used additional proteomics databases to validate the causal relationships of identified proteins, which greatly reduced the chance of false positives. However, there are various constraints in our research. First, the pQTL data of the proteins we selected were derived from plasma and may not fully reflect the specific changes in retinal tissue. Future studies should use ocular tissue-derived proteins to further explore drug targets. Secondly, in our study, the number of plasma proteins considered as exposure is limited. As more GWAS data on plasma proteins are developed in the future, more comprehensive MR study should be performed to further explore novel drug targets for DR. Thirdly, the identified prioritized proteins were found to have just one cis-acting SNP, which limited further verifications. Fourthly, our analysis was conducted specifically on populations of European descent, making it challenging to extrapolate the findings to other ancestral backgrounds. Finally, despite conducting numerous validations and sensitivity analyses, it is crucial to provide a thorough explanation of the observed findings, as they are indicative rather than definitive in determining the causal identities of these proteins for DR. Thus, additional research is necessary to validate these findings, such as studies in in-vitro cell lines, animal models and clinical samples.

## Conclusion

To summarize, our research showed a cause-and-effect connection between genetically determined levels of CFH, CFHR4, and B3GNT8 plasma proteins and DR. The discovery implies that these proteins hold potential as drug targets in the process of developing drugs to treat DR. However, further research is necessary to fully understand the functions of these proteins in the development and progression of DR.

### Supplementary Information

Supplementary Tables.

## Data Availability

The original contributions presented in the study are included in the article/supplementary material. Further inquiries can be directed to the corresponding author.
